# The application of CRISPR/Cas9 in hairy roots to explore the functions of *AhNFR1* and *AhNFR5* genes during peanut nodulation

**DOI:** 10.1186/s12870-020-02614-x

**Published:** 2020-09-07

**Authors:** Hongmei Shu, Ziliang Luo, Ze Peng, Jianping Wang

**Affiliations:** 1 Institute of Industrial Crops, Jiangsu Academy of Agricultural Sciences, Nanjing, 210014 China; 2 Agronomy Department, University of Florida, Gainesville, FL 32610 USA

**Keywords:** CRISPR/Cas9, Mutants, Nod factor receptor (*NFR*) genes, Nodulation, Peanut, Symbiosis

## Abstract

**Background:**

Peanut is an important legume crop growing worldwide. With the published allotetraploid genomes, further functional studies of the genes in peanut are very critical for crop improvement. CRISPR/Cas9 system is emerging as a robust tool for gene functional study and crop improvement, which haven’t been extensively utilized in peanut yet. Peanut plant forms root nodules to fix nitrogen through a symbiotic relationship with rhizobia. In model legumes, the response of plants to rhizobia is initiated by Nod factor receptors (NFRs). However, information about the function of *NFRs* in peanut is still limited. In this study, we applied the CRISPR/Cas9 tool in peanut hairy root transformation system to explore the function of *NFR* genes.

**Results:**

We firstly identified four *AhNFR1* genes and two *AhNFR5* genes in cultivated peanut (*Tifrunner*). The gene expression analysis showed that the two *AhNFR1* and two *AhNFR5* genes had high expression levels in nodulating (Nod+) line E5 compared with non-nodulating (Nod-) line E4 during the process of nodule formation, suggesting their roles in peanut nodulation. To further explore their functions in peanut nodulation, we applied CRISPR technology to create knock-out mutants of *AhNFR1* and *AhNFR5* genes using hairy root transformation system. The sequencing of these genes in transgenic hairy roots showed that the selected *AhNFR1* and *AhNFR5* genes were successfully edited by the CRISPR system, demonstrating its efficacy for targeted mutation in allotetraploid peanut. The mutants with editing in the two *AhNFR5* genes showed Nod- phenotype, whereas mutants with editing in the two selected *AhNFR1* genes could still form nodules after rhizobia inoculation.

**Conclusions:**

This study showed that CRISPR-Cas9 could be used in peanut hairy root transformation system for peanut functional genomic studies, specifically on the gene function in roots. By using CRISPR-Cas9 targeting peanut *AhNFR* genes in hairy root transformation system, we validated the function of *AhNFR5* genes in nodule formation in peanut.

## Background

Cultivated peanut (*Arachis hypogaea* L.) is a leguminous crop with great economic values mainly for oil and food production. Cultivated peanut is an allotetraploid with two sub-genomes, A and B (AABB genome, 2*n =* 4x = 40). The two sub-genomes were presumably derived from two diploid ancestral species *A. duranensis* and *A. ipaensis* [1]. The genomes of the two ancestral species and two cultivated peanut cultivars (*Tifrunner and Shitouqi*) were fully sequenced and publicly available [1–3]. These reference genomes greatly facilitate peanut molecular and genetic studies. To characterize genes’ functions in peanut genome, engineered gene overexpression and RNA interference (RNAi) have been widely used [4–6]. However, the gene overexpression can cause side effects for the organism and the RNAi knockdown cannot eliminate the function of remaining proteins, hence limited their ability for gene function characterization. There is a need to update the arsenal of gene functional analysis for peanut genetic study.

The CRISPR/Cas9 (clustered regularly interspaced short palindromic repeats/CRISPR-associated protein 9) system has successfully demonstrated precise gene editing in many plant systems [7]. CRISPR/Cas-mediated gene knockout, replacement, and insertion provide simple and efficient approaches for gene functional studies, plant biology, and precision plant breeding [8, 9]. The application of CRISPR/Cas9 in plants empowered plant breeders to control the target genes, which provides a great resource for rapid crops improvement [8, 9]. It’s believed that CRISPR/Cas has the potential to enhance global food security and sustainable agriculture. However, there is little knowledge of the application of CRISPR/Cas9 in peanut.

The nitrogen fixing root-nodule symbiosis in peanut and other legume plants allows them to grow well in soil without or lack of nitrogen fertilizer input and produce protein-rich seeds. Most legumes including model legumes establish root-nodule symbiosis through the “root hair” infection. The plant root exudates such as flavonoids specifically induce the transcription of nodulation genes (*Nod, Nol, Noe*) [10, 11], which are involved in the synthesis of nodulation factors (Nod factors, NFs) in rhizobia. NFs are recognized by membrane lysin motif (LysM) receptor-like kinases (LYK/LYR) of host root epidermal cells, and subsequently stimulate the signal transduction in plant roots to facilitate the rhizobial infection through infection thread (IT) formation and nodule primordia formation [12, 13]. The peanut form nodules predominantly with NF-producing *Bradyrhizobium* strains, but NF mutant *Bradyrhizobium* was reported to induce nodules in peanut [14, 15]. The mode of rhizobial invasion in peanut is known as the “crack-entry” [16, 17], which is different from the “root hair” infection path. The rhizobia enter the root through the middle lamella between adjacent axillary hair cells and invade into the cortex intercellularly. The large basal cells infected by the rhizobia divide repeatedly to form determinate nodules.

The recognition of NF from compatible rhizobia is a crucial step for root nodule symbiosis. Two NF receptor (*NFR*) genes or their orthologs were identified and characterized in several legumes with root hair infection [18–24]. These *NFR* genes were named *NFR1* and *NFR5* in *L. japonicus*, both encoding LysM-type serine/threonine receptor kinases [19]. The response of legumes to rhizobial infection is initiated by NFRs, and symbiotic receptor kinase (SYMRK) amplifies the NF-perceived signal [14] which activates downstream symbiotic signaling pathway to start the nodule morphogenesis [14, 25, 26].

In peanut, two putative NFRs (AhNFR1 and AhNFP) were identified [27], which are the orthologs of LjNFR1 and LjNFR5, respectively. The predicted protein of AhNFP had similar molecular features to both soybean GmNFR5α and GmNFR5β [27]. In soybean, overexpression of *GmNFR5α*, *GmNFR5β* and *GmNFR1α* in Nod- mutants could recover Nod+ phenotypes [22, 28], and overexpression of *GmNFR1α* also increased the nodule number per plant. The NFR proteins encoded by these two genes in *Lotus japonicus* form a heteromeric receptor complex to initiate downstream signaling [29]. Our previous reports [26, 30] indicated that the peanut *NFR5* ortholog may play a role in recognizing nod factors. However, Karmakar et al. [15] considered that the peanut orthologs of *NFR1* and *NFR5* might not be the key genes for symbiosis establishment, because the expression levels of lipochitooligosaccharide (LCO)-binding receptor gene (*LYR3)* and exopolysaccharide (EPS) receptor gene (*EPR3)* were much higher than that of the classical *NFRs* (*NFR1* and *NFR5*) in peanut. In this study, we conducted hairy root mediated CRISPR knockout of peanut *AhNFR1* and *AhNFR5* genes to characterize their functions in nodulation symbiosis. The results not only approved that CRISPR/Cas9 in coupled with hairy root transformation system is a rapid approach in characterizing gene functions in roots, but also improved our understanding of the *NFR* genes’ function in peanut nodulation.

## Results

### Retrieving and comparing the gene sequences of *AhNFR1s* and *AhNFR5s*

Using the coding DNA sequences (CDS) of *GmNFR1* (DQ219806) and *GmNFR5* (NM_001354196) as queries, four *AhNFR1* genes (two on A sub-genome named *AhNFR1A1* and *AhNFR1A2*, two on B sub-genome named *AhNFR1B1* and *AhNFR1B2*) and two *AhNFR5* genes (*AhNFR5A* on A sub-genome and *AhNFR5B* on B sub-genome) were identified from the peanut genomes at PeanutBase (Table 1). The two *AhNFR5* genes had no intron while the four *AhNFR1* genes had multiple introns (Table 1). Among the four *AhNFR1* genes, the length of *AhNFR1A1* sequence was longer than the other three *AhNFR1* genes due to its additional number of introns and expanded exons. The DNA sequence homology between *AhNFR5A* and *AhNFR5B* was 98%; the homology between *AhNFR1A1* and *AhNFR1B1* was 96%; the homology between *AhNFR1A2* and *AhNFR1B2* was 99% (Additional file 1: Fig. S1).

**Table 1:** The summary of *AhNFR1* and *AhNFR5* genes identified in peanut genome

Genes	Gene model name	Orthologous gene in A or B genome	Position in the genomes	CDS length (bp)	No. of introns
*AhNFR5A*	Arahy.VID2UW	Aradu.AXP1M	Arahy.05:9587654 ~ 9,589,441 (+ strand)	1788	0
*AhNFR5B*	Arahy.A8RCAK	Araip.NL2P7	Arahy.15:9907797 ~ 9,909,581 (+ strand)	1785	0
*AhNFR1A1*	Arahy.63XNPZ	Aradu.P4UQH	Arahy.07:5753891 ~ 5,769,002 (− strand)	5220	35
*AhNFR1B1*	Arahy.IVY8DS	Araip.YG8RE	Arahy.17:7510512 ~ 7,514,680 (− strand)	1959	12
*AhNFR1A2*	Arahy.MX792F	Aradu.8UK90	Arahy.04:1275786 ~ 1,283,663 (+ strand)	1869	11
*AhNFR1B2*	Arahy.SA9NCH	Araip.8R7CF	Arahy.14:1845319 ~ 1,852,888 (+ strand)	2130	11

**Fig. 1:**
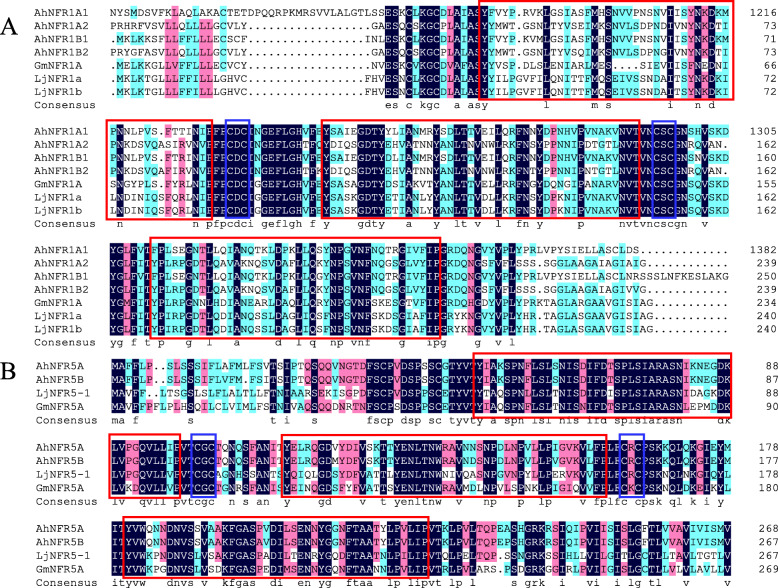
Comparison of partial amino acid sequences of NFRs in *Arachis hypogaea*, *Glycine max*, and *Lotus japonicus*. **a** Amino acid sequence of NFR1. **b** Amino acid sequence of NFR5. The sequences in red boxes are the extracellular LysM domains of NFRs. The sequences in blue boxes are the CXC motifs. Protein IDs were: GmNFR1A (ABB30246.1), GmNFR5A (NP_001341125.1), LjNFR1a (CAE02591.1), LjNFR1b (CAE02592.1), LjNFR5–1 (CAE02597.1)

The functional structures of the six putative peanut NFR protein sequences were similar to those reported orthologs in other legumes [31, 32]. They had three extracellular LysM domains, typical CXC (Cysteine-any amino acid-Cysteine) motifs in the interspaces domains between LysM1-LysM2 and LysM2-LysM3 [27] (Fig. 1).

**Fig. 2:**
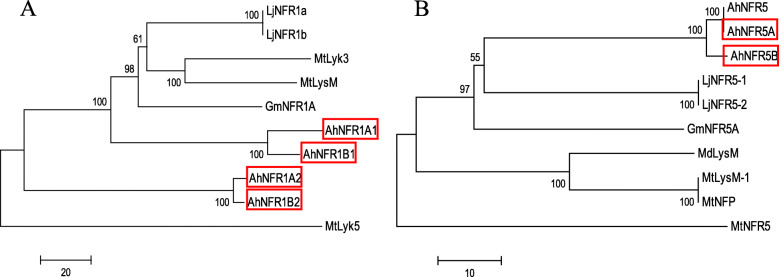
The unrooted neighbor joining phylogenetic tree of NFRs in cultivated peanut and other legumes. **a NFR1. b NFR5.** The red boxes highlighted the NFRs orthologs we identified in PeanutBase. Other protein IDs were: AhNFR5 (ANS10208.1), GmNFR1A (ABB30246.1), GmNFR5A (NP_001341125.1), LjNFR1a (CAE02591.1), LjNFR1b (CAE02592.1), LjNFR5–1 (CAE02597.1), LjNFR5–2 (CAE02598.1), MdLysM (AEN71536.1), MtLyk3 (AAQ73159.1), MtLyk5 (CAN88846.1), MtLysM (CAN88845.1), MtLysM-1 (CAO02950.1), MtNFP (ABF50224.1), MtNFR5 (AET03984.1)

Phylogenetic analysis based on protein sequences (Fig. 2) showed that AhNFR5A and AhNFR5B were closely related to LjNFR5–1/2 and GmNFR5A; four AhNFR1 proteins are the orthologs of LjNFR1 and GmNFR1A.

### Investigating the expression patterns of *AhNFR1s* and *AhNFR5s* during rhizobial infection

**Fig. 3:**
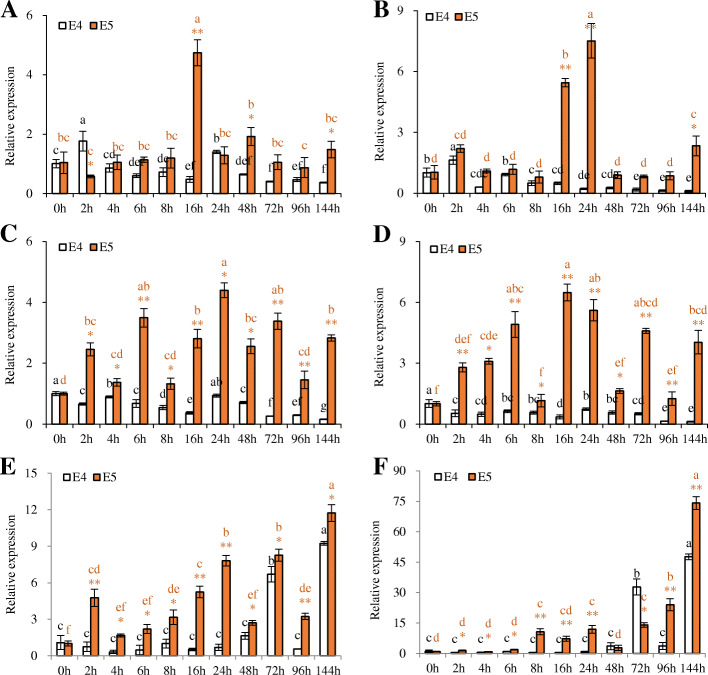
Expression patterns of *AhNFR* genes in roots of Nod- E4 and Nod+ E5 inoculated with rhizobia Lb8. The time points included 0 h, 2 h, 4 h, 6 h, 8 h, 16 h, 24 h, 48 h, 72 h, 96 h and 144 h for *AhNFR1A1* (**a**), *AhNFR1B1* (**b**), *AhNFR1A2* (**c**), *AhNFR1B2* (**d**), *AhNFR5A* (**e**), and *AhNFR5B* (**f**). Asterisks indicate significant differences between E4 and E5 as determined by student’s t-test (*, *p* < 0.05; **, *p* < 0.01). Letters on columns indicate the significantly different (*p* < 0.05) groups within E4 (black letters) or within E5 (orange letters) after post-hoc comparison

To investigate the temporal gene expression patterns of these genes in Nod- E4 and Nod+ E5 after rhizobia inoculation, quantitative real time-PCR (q-PCR) was conducted. The six genes had different expression patterns in roots of Nod- E4 and Nod+ E5 after rhizobia inoculation (Fig. 3). Among the six putative peanut *AhNFR* genes, the expression level of the four *AhNFR1* genes was not induced in Nod- E4 after rhizobia inoculation, but they were induced in Nod+ E5 after inoculation. Both *AhNFR1A1* and *AhNFR1B1* were induced in Nod+ E5, but only at 16 h after inoculation (HAI) for *AhNFR1A1* and at 16 and 24 HAI for *AhNFR1B1* (Fig. 3a and b). However, *AhNFR1A2* and *AhNFR1B2* were significantly induced in Nod+ E5 at all time points tested including early and late infection stages. The results suggested that among the four *AhNFR1* genes, *AhNFR1A2* and *AhNFR1B2* might play major roles in rhizobial infection and nodule organogenesis when comparing with *AhNFR1A1* and *AhNFR1B1*, because their expression levels are much more significantly different between Nod- E4 and Nod+ E5 (Fig. 3c and d). The expressions of *AhNFR5A* and *AhNFR5B* were only induced in Nod- E4 at 72 and 144 HAI, but these two genes were induced in Nod+ E5 throughout the infection stages. The significantly high expression levels of the two *AhNFR5* genes in Nod+ E5 suggesting they were related with rhizobial infection and nodule organogenesis (Fig. 3e and f).

In addition, the CDSs of *AhNFR1A2*, *AhNFR1B2*, *AhNFR5A* and *AhNFR5B* of Nod- E4 and Nod+ E5 were checked (data not shown). The sequence comparisons showed that the four genes were all identical between Nod- E4 and Nod+ E5, and they were also identical to the DNA sequences obtained in PeanutBase. The results indicated that none of the four *AhNFR* genes were mutated in Nod- E4 line.

Therefore, two *AhNFR1* genes *(AhNFR1A2* and *AhNFR1B2)* and two *AhNFR5* genes *(AhNFR5A* and *AhNFR5B)* were selected to study whether these genes are required for nodule formation in peanut using CRISPR/Cas9 system.

### Target site selection and construction of the sgRNA: Cas9 expression vector

Two single guide RNAs (sgRNAs) named NFR1AB1 and NFR1AB2 were designed to target both *AhNFR1A2* and *AhNFR1B2* genes, but at different sites. Accordingly, p201G/Cas9:NFR1AB1 + NFR1AB2 vector harboring the both sgRNAs (NFR1AB1 and NFR1AB2) were inserted into one plasmid p201G/Cas9 to target both *AhNFR1A2* and *AhNFR1B2* genes for knocking out (Table 2). For *AhNFR5* genes, three sgRNAs were designed (Table 2). Among them, one sgRNA (NFR5B) was designed to target *AhNFR5B* gene and the other two sgRNAs (NFR5AB1 and NFR5AB2) were designed to target both *AhNFR5A* and *AhNFR5B* genes at two different sites. Therefore, p201G/Cas9:NFR5AB1 + NFR5AB2 vector targeting both *AhNFR5A* and *AhNFR5B* genes and p201G/Cas9: NFR5B vector targeting only *AhNFR5B* gene were constructed, separately (Table 2, Additional file 1: Fig. S2).

**Table 2:** Target sequences of sgRNAs designed in this study

sgRNAs	Target sequence (5′-3′)	Targeted genes	Constructs (plasmid:sgRNAs)
NFR1AB1	TCTAGCTTCCTACTACATG	*AhNFR1A2* and *AhNFR1B2*	p201G/Cas9:NFR1AB1 + NFR1AB2
NFR1AB2	TTACCGTTAACTGCTCCTG		
NFR5AB1	GCAAGTAACATAAAGAATG	*AhNFR5A* and *AhNFR5B*	p201G/Cas9:NFR5AB1 + NFR5AB2
NFR5AB2	CTTGGGGGCACAGTTTACA		
NFR5B	ACTCAACCCGAGCCTTCACA	*AhNFR5B*	p201G/Cas9:NFR5B

### Characterizing transgenic hairy roots

#### AhNFR1 gene

To validate the exogenous T-DNA insertion into transgenic hairy roots, only Green fluorescent protein (GFP) positive hairy roots, transformed with p201G/Cas9:NFR1AB1 + NFR1AB2 vector, were subjected to sequencing validation. 12 independent *AhNFR1* transgenic events showing GFP positive, named as H1 to H12. To evaluate the DNA editing on targeted *AhNFR1* genomic regions, amplicons covering the two target sites (NFR1AB1 and NFR1AB2) were evaluated and further cloned and sequenced from the 12 hairy roots.

Amplicons from 7 out of 12 samples (H3, H4, H6, H8, H9, H11 and H12) had only one PCR band, with the same size (~ 600 bp) as the wild type (WT). Whereas the rest five samples (H1, H2, H5, H7, H10) had two PCR bands, and the sizes of them were around 300 and 600 bp (Additional file 1: Fig. S3). Based on our original design, the size of the expected main PCR product was 569 bp, and the designed cleavage length was about 300 bp. Thus, this result demonstrated that the precise cleavage events probably occurred in our designed genomic regions of *AhNFR1* genes. To further validate whether the changed PCR size was derived from CRISPR/Cas9-caused genomic truncation, we randomly picked 20 positive clones generated from each band of PCR products of each transgenic *AhNFR1* peanut hairy root sample for Sanger sequencing.

**Fig. 4:**
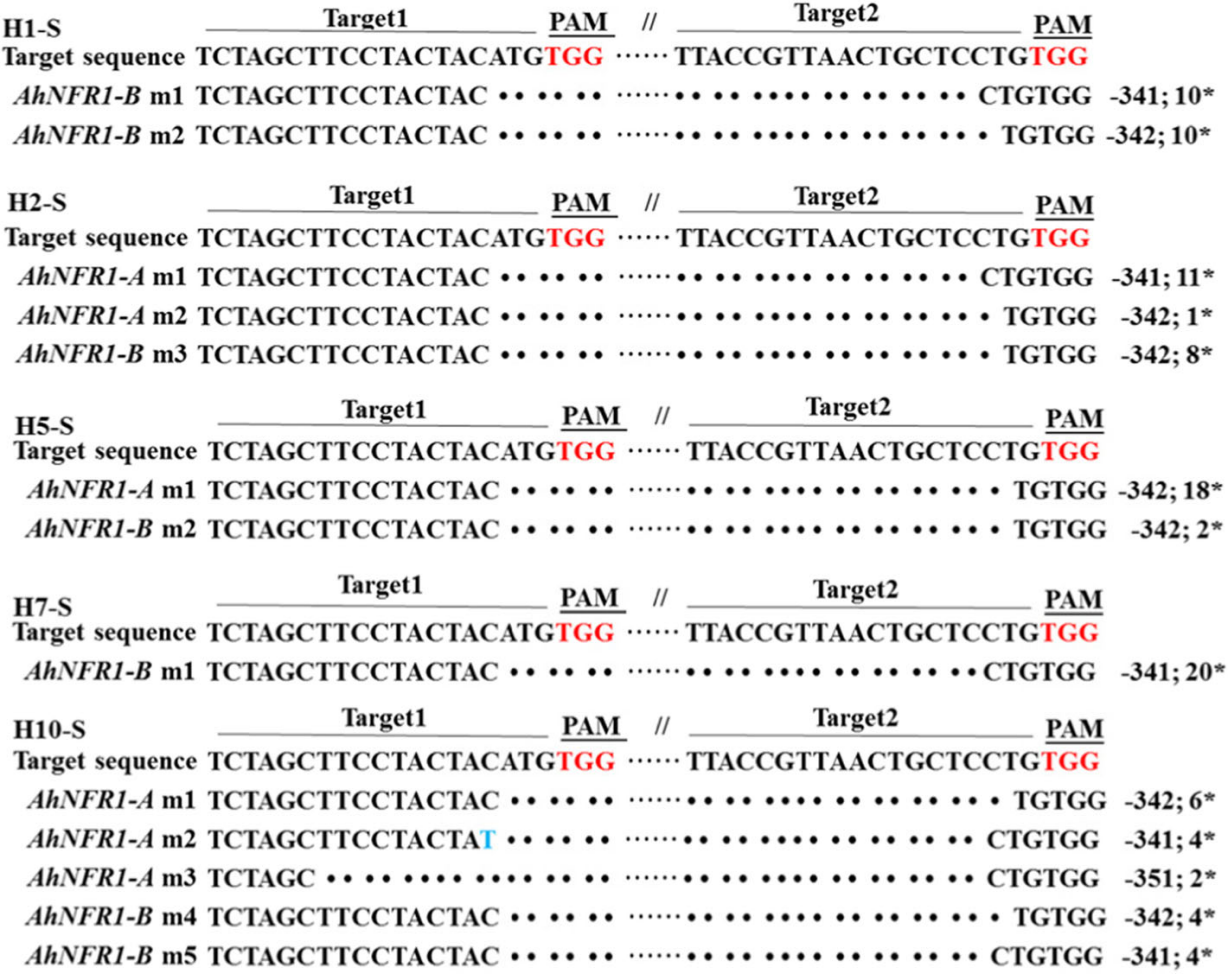
Validation of CRISPR/Cas9-induced long fragment deletions at both *AhNFR1A2* and *AhNFR1B2* DNA sequence sites. H1-S, H2-S, H5-S, H7-S, and H10-S: alignment of genomic sequences cloned from the truncated PCR products using DNA samples H1, H2, H5, H7, H10 of transgenic *AhNFR1* hairy roots as templates. Target 1 and target 2 represent target NFR1AB1 and target NFR1AB2. Wild-type sequences are in black, deletions are shown as dots, and insertions are shown in blue. ‘-’ on the right represent nucleotide deletions. PAM, protospacer adjacent motif

The sequencing results of samples H1, H2, H5, H7 and H10 demonstrated that all 100 smaller sequences (20 clones for each sample) were truncated versions of *AhNFR1* genomic sequences from NFR1AB1 DNA site to NFR1AB2 DNA site (Fig. 4). The cleavage DNA length was mostly − 342 and − 341 bp (Table 3). Thus, the CRISPR/Cas9 genome editing system succeeded in generating long DNA fragment deletions on the selected genomic region in peanut genomes.

**Table 3:** Percentages of CRISPR/Cas9-caused *AhNFR1* truncated events

Samples	Genes	Rate of different nucleotide deletion (%)	
		-341 bp	−342 bp
H1	*AhNFR1A2*	0	0
	*AhNFR1B2*	50	50
H2	*AhNFR1A2*	92	8
	*AhNFR1B2*	0	100
H5	*AhNFR1A2*	0	100
	*AhNFR1B2*	0	100
H7	*AhNFR1A2*	0	0
	*AhNFR1B2*	0	100
H10	*AhNFR1A2*	33	50
	*AhNFR1B2*	50	50

**Fig. 5:**
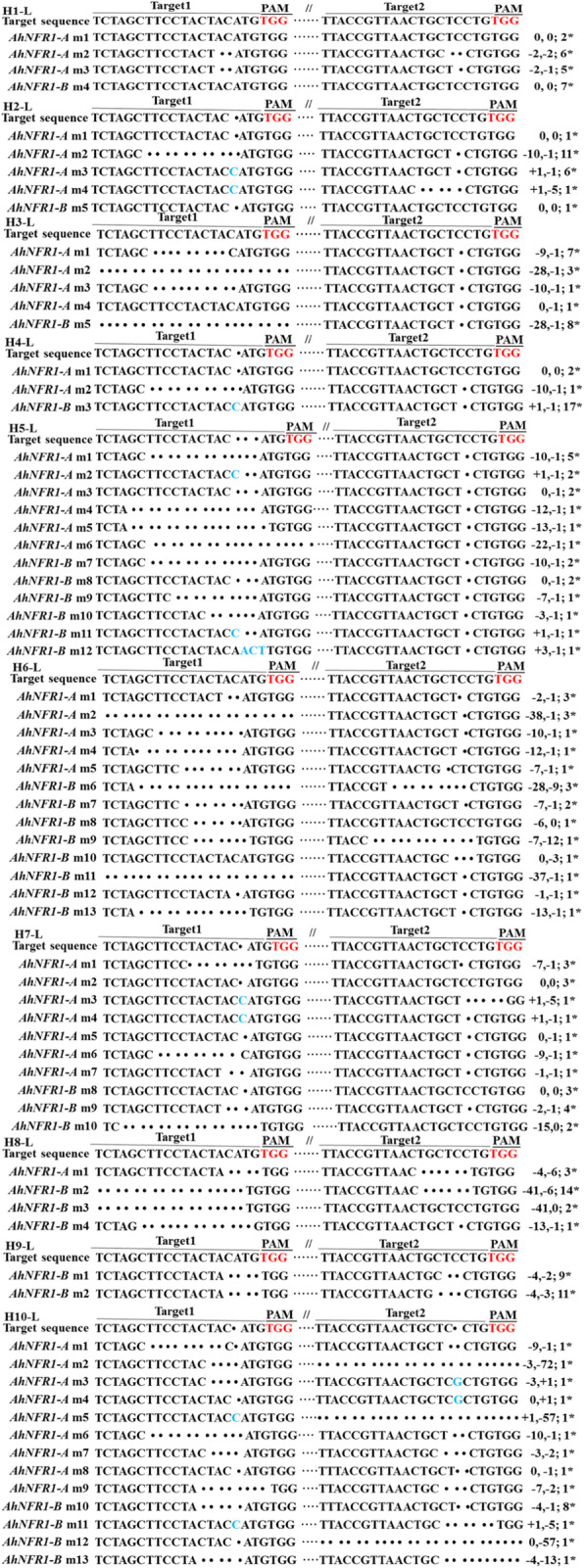
Validation of CRISPR/Cas9-induced mutations at both *AhNFR1A2* and *AhNFR1B2* DNA sequence sites. H1-L ~ H10-L: alignment of genomic sequences cloned from the long PCR products using DNA samples H1 ~ H10 of transgenic *AhNFR1* hairy roots as templates. Target 1 and target 2 represent target NFR1AB1 and target NFR1AB2. Wild-type sequences are in black, deletions are shown as dots, and insertions are shown in blue. ‘-’, ‘+’ and ‘0’ on the right represent nucleotide deletions, insertions and no mutation, respectively

For the 12 samples with longer PCR product of 569 bp, 240 positive clones (20 clones for each sample’s PCR product) were randomly picked for Sanger sequencing analyses. The results showed no modifications at the target genes in hairy roots H11 and H12 (data not shown). However, various types of nucleotide insertion and deletion mutations at target NFR1AB1 genomic sites were observed on the other 10 samples, and mostly − 1 bp deletion at target NFR1AB2 genomic site (Fig. 5) (Additional file 1: Table S1). Except for sample H9, in the rest nine samples, examined target NFR1AB1 and NFR1AB2 genomic sites of some clones still kept unedited, the same as the wild type DNA sequence. The results suggested that among the genome knockout transgenic *AhNFR1* events, mosaicism was observed in each transgenic event, which may disturb later phenotypic analysis [33]. The phenotyping of transgenic *AhNFR1* hairy roots after rhizobia inoculation showed that all the positively transformed hairy roots with genome editing produced nodules except for samples H4 and H6. Therefore, we considered that the modification of *AhNFR1A2* and *AhNFR1B2* genes identified in this study did not affect the nodule formation.

#### *AhNFR5* gene

To validate the exogenous T-DNA insertion in *AhNFR5* transgenic hairy roots transformed with p201G/Cas9:NFR5AB1 + NFR5AB2 vector (targeting both *AhNFR5A* and *AhNFR5B* genes), 10 independent transgenic events showing GFP positive, named as F1 to F10 were screened. To evaluate the DNA editing occurred at the targeting *AhNFR5A* and *AhNFR5B* genomic regions of the 10 events, amplicons covering the two target sites (NFR5AB1 and NFR5AB2), were evaluated and further cloned and sequenced.

**Fig. 6:**
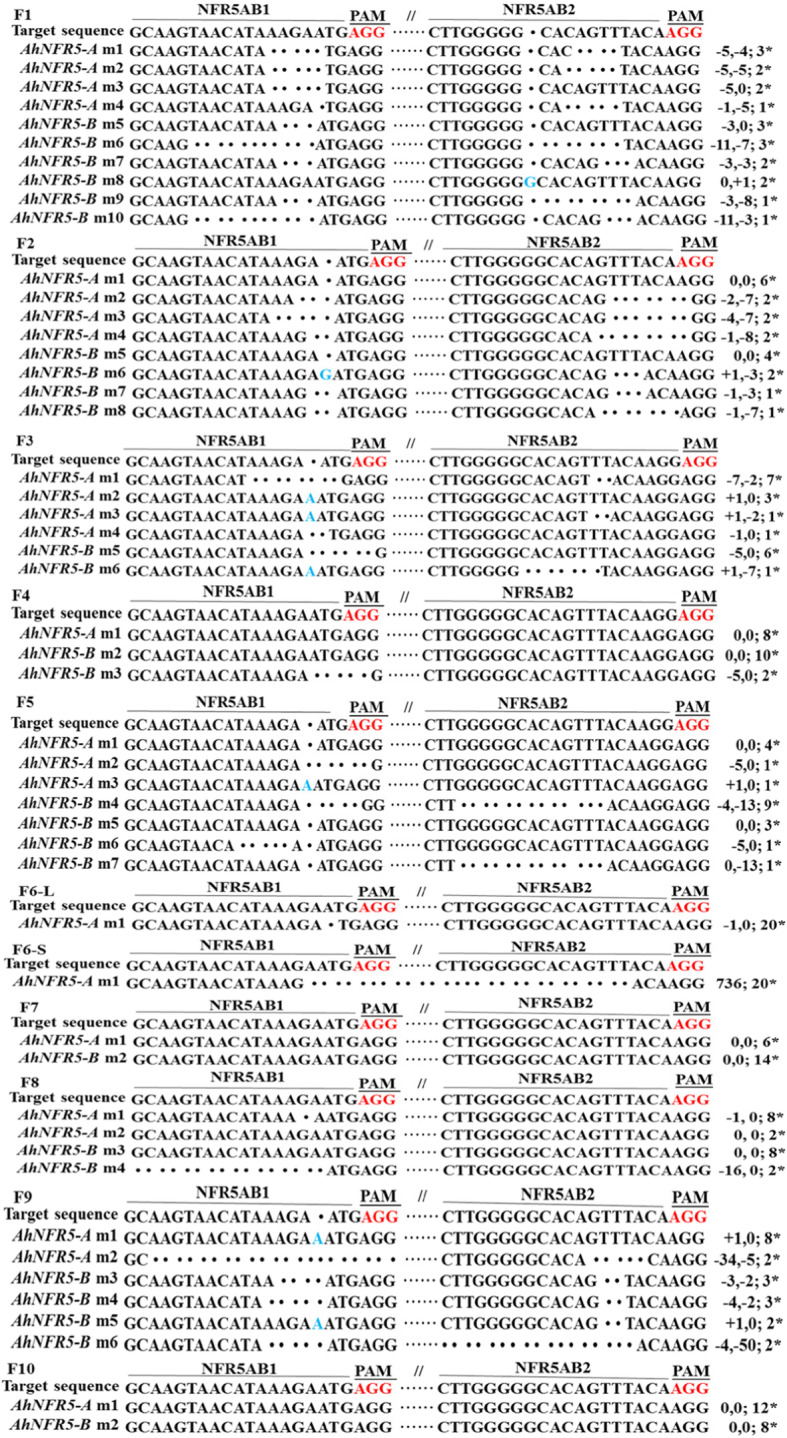
Validation of CRISPR/Cas9-induced mutations at both *AhNFR5A* and *AhNFR5B* DNA sequence sites. F1 ~ F10: alignment of genomic sequences cloned from the PCR products using DNA samples F1 ~ F10 of transgenic *AhNFR5* hairy roots as templates. NFR5AB1 and NFR5AB2 represent two targets. Wild-type sequences are in black, deletions are shown as dots, and insertions are shown in blue. ‘-’, ‘+’ and ‘0’ on the right represent nucleotide deletions, insertions and no mutation, respectively

Except for sample F6, the PCR products in nine transgenic hairy roots had only one PCR band (Additional file 1: Fig. S4). We also selected 20 clones of the PCR product of each band for sequencing. The PCR products in hairy root F6 had two bands and the sequencing results showed that not only long sequence (736 bp) deletion but also 1 bp deletion at target NFR5AB1 site occurred (Fig. 6) (Additional file 1: Table S2). Among the 10 hairy roots, there were no modifications detected in *AhNFR5* gene sequence from samples F7 and F10. In the other eight samples, the sequences of all clones of sample F1, F3, F6 and F9 at target NFR5AB1 or target NFR5AB2 site were edited, while some clones of other samples at the two target sites were not edited. Mosaicism was observed in the 8 transgenic *AhNFR5* events.

The sequence mutations on *AhNFR5* hairy roots caused Nod- phenotype of sample F1, F3, F6 and F9 (Table 4). The sequencing and phenotype results indicated that when *AhNFR5A* and *AhNFR5B* genes’ sequence of all clones from one transgenic *AhNFR5* event were all edited, no nodule could be formed. Therefore, we concluded that *AhNFR5A* and *AhNFR5B* genes were the key genes required for peanut nodule formation.

**Table 4:** Mutation genotype and nodulation phenotype of independent *AhNFR5*-edited hairy root

Samples	Mutation at target 5AB1 site		Mutation at target 5AB2 site		GFP positivity	Nodule phenotype
	At	Bt	At	Bt		
F1	−5,-1,	−3, −11	−4,-5,n	−3,−7,-8,+ 1	+	–
F2	n,-2,-4,-1	n,-1,+ 1	n,-7,-8	n,-3,-7	+	+
F3	-7,+ 1,-1	−5,+ 1	−2,n	n,-7	+	–
F4	n	n,-5	n	n	+	+
F5	n,-5,+ 1	n,-4,-5	n	n,−13	+	+
F6	-1,-736	/	n,-736	/	+	–
F7	n	n	n	n	+	+
F8	n,-1	n,-16	n	n	+	+
F9	+ 1,−34	-3,-4,+ 1	−5,n	−2,n,-50	+	–
F10	n	n	n	n	+	+

‘At’ represents target sequence in *AhNFR5A* gene; ‘Bt’ represents target sequence in *AhNFR5B* gene. ‘-’, ‘+’ and ‘n’ in the left four columns represent nucleotide deletions, insertions and no mutation respectively. ‘-’ and ‘+’ in the right two columns represent negative and positive

**Fig. 7:**
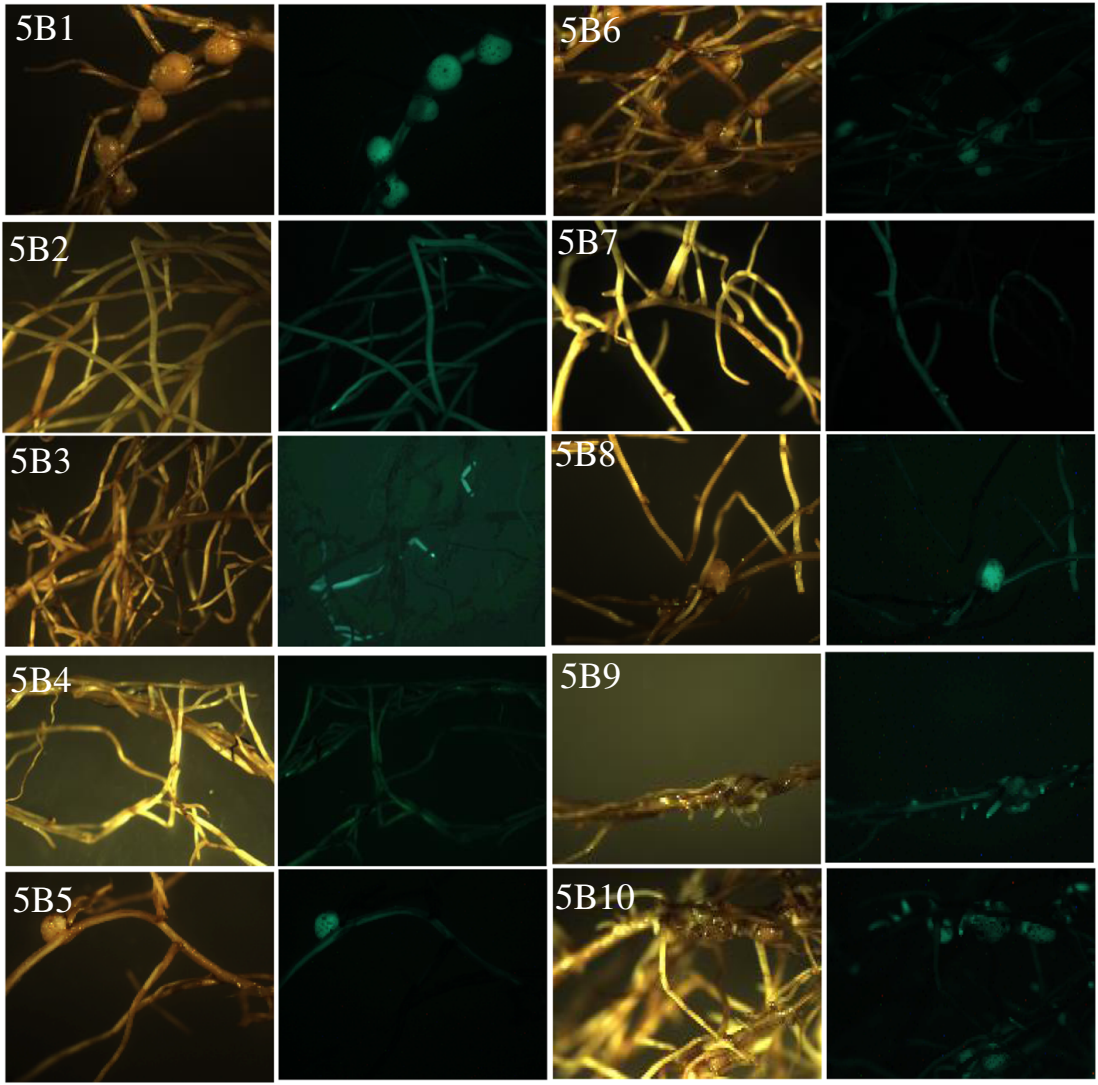
Nodulation phenotypes of transgenic *AhNFR5B* hairy roots. 5B1 ~ 5B10 are ten independent events. The left images were hairy root under white light, and the right images were hairy roots under fluorescence light

To validate the exogenous T-DNA insertion in *AhNFR5B* transgenic hairy roots transformed with p201G/Cas9:NFR5B vector (targeting only *AhNFR5B* gene), DNAs were extracted from 10 independent transgenic events. The PCR products of *AhNFR5B* in 10 transgenic hairy roots had only one PCR band (Additional file 1: Fig. S5). As shown in Table 5, 10 independent transgenic events of *AhNFR5B* samples (5B1 ~ 5B10) were analyzed. The sequencing results showed that there was no modification detected in A sub-genome from the 10 hairy roots, indicating that there was no mismatched nucleotide with NFR5B-sgRNA. No sequence editing was detected in samples 5B1, 5B5, 5B6, 5B8 and 5B10. However, various types of nucleotide insertion and deletion mutations were detected in the rest five samples: 5B2, 5B3, 5B4, 5B7 and 5B9. The phenotype results of transgenic *AhNFR5B* hairy roots (Fig. 7) showed that samples 5B2, 5B3, 5B4, 5B7 and 5B9 had no nodule, but other unedited hairy roots had nodules. Among the five no-nodule hairy roots, *AhNFR5B* genes’ sequence of all clones from samples 5B2, 5B3, and 5B9 were all edited, but *AhNFR5B* genes’ sequences of all clones from samples 5B4 and 5B7 were partly edited. Combining the results of *AhNFR5* transgenic hairy roots transformed with p201G/Cas9: NFR5AB1 + NFR5AB2 vector, these results showed that *AhNFR5B* genes’ sequence of all clones from one transgenic event were all edited, which could cause non-nodulation phenotype. However, if *AhNFR5B* genes’ sequence of clones from one transgenic event were partly edited, the phenotype remained uncertain.

**Table 5:**
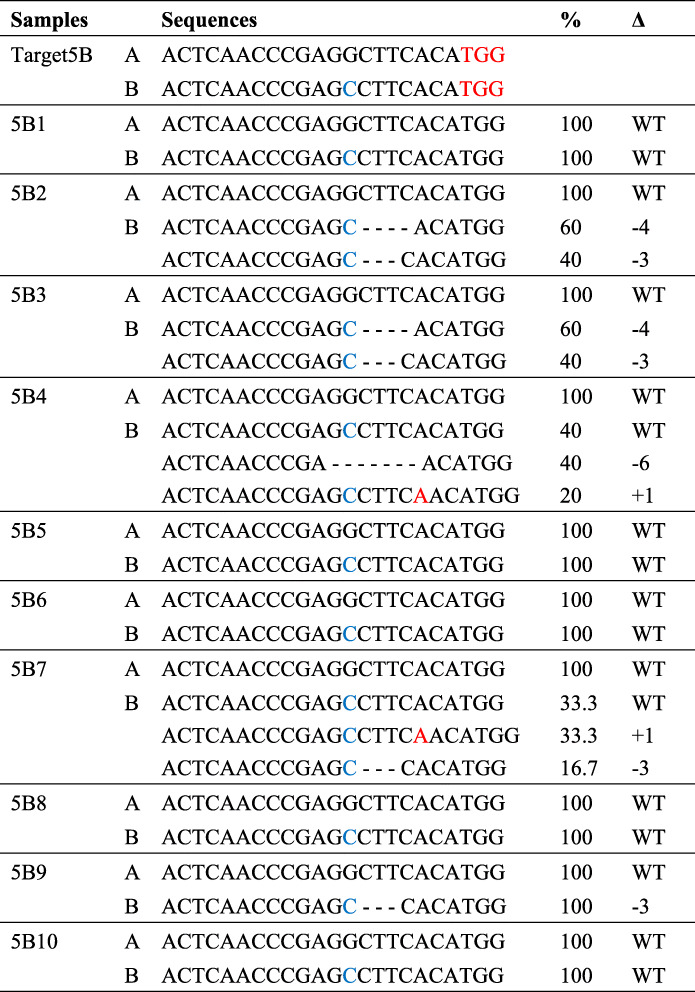
Sequence analyses of CRISPR/Cas9-induced mutations at *AhNFR5B* DNA sequence site of transgenic *AhNFR5B* hairy roots

Sequence confirmation of nucleotide deletion and insertion mutations in peanut transgenic *AhNFR5B* samples. ‘WT’ represents wild type. Wild-type sequences are in black, difference between alleles is in blue, deletions are shown as dashes, and insertions are shown in red. Percentages next to sequences indicate the number of clones with sequence over the number of total clones sequenced. **Δ** is the base difference comparing with WT.

## Discussion

### CRISPR system

Peanut is an important economic crop. With the achievements in peanut genome researches and whole genome-wide gene identification studies, the peanut community urgently needs a highly efficient and cost-effective CRISPR/Cas9 system for gene function studies. To establish the CRISPR system for root-related studies in peanut, the p201G/Cas9 vector, which showed high editing efficiency in soybean [34], was selected for this study. To examine the qualification for effective CRISPR/Cas9 genomic editing in allotetraploid peanut genome, two sgRNAs (NFR1AB1 and NFR1AB2) for *AhNFR1A2* and *AhNFR1B2* and two sgRNAs (NFR5AB1 and NFR5AB2) for *AhNFR5A* and *AhNFR5B* designed in identical genomic regions between the homologous copies at A sub-genome and B sub-genome, were employed to examine the efficiency of this genome editing system in allotetraploid peanut. The various type of editing results of both targeted genes indicated that p201G/Cas9 CRISPR vector had high editing efficiency, since the long genomic fragment deletions required high Cas9-sgRNA-complex activity to ensure the two designed cleavage sites be effectively recognized and cleaved simultaneously [35, 36].

A single specific sgRNA (NFR5B) was designed for *AhNFR5B* gene. The sequencing results showed that *AhNFR5B* gene was edited in 50% of GFP-positive transgenic *AhNFR5B* hairy roots. However, *AhNFR5A* and *AhNFR5B* genes were edited in 80% of transgenic *AhNFR5* hairy roots with two sgRNAs (NFR5AB1 and NFR5AB2) targeting both *AhNFR5A* and *AhNFR5B* genes. This result suggested that the editing efficiency of one sgRNA in CRISPR vector was lower than that of two sgRNAs. In another word, two or more sgRNAs in one CRISPR vector could increase the gene editing efficiency.

In summary, our study provided a successful example of using CRISPR/Cas9 system for highly efficient genome editing in allotetraploid peanut, which can be widely applied to generate mutations for other functional genomics studies or crop improvement in peanut.

A. *rhizogenes* mediated hairy root production has been utilized as a powerful tool in peanut to discover gene function and study root biology [5, 6, 37]. It is a highly efficient technique for generating composite plants composed of transgenic roots and wild-type shoot. In this study we constructed the CRISPR/Cas9 system in hairy roots to explore gene function during nodule formation. However, the hairy root transformation system may have its limitations to study gene functions by CRISPR/Cas9 technology. Particularly, mosaicism was observed in each transgenic event, which might disturb later phenotypic analysis. Mosaic mutations are a major challenge of CRISPR/Cas9 editing system [38]. Previous research [33] inferred that mosaicism could be eliminated during the later stage of selection. However, hairy root transformation system could not generate offspring. Due to the possible mosaicism, hairy root transformants from CRISPR-Cas9 experiment would generate Nod-, Nod+ or Nod−/Nod+ sectors. Therefore, in the current study, the clear Nod- phenotype observed from the knockout events (Fig. 7) provided us certain confidence in the knockout experiments. Future improvement of this gene editing system can be achieved by applying the stable embryogenesis transformation systems.

### The functions of *AhNFR* genes during nodule formation

In a common symbiotic signaling pathway characterized in model legume species, the first step is the recognition of NFs by LYK. There are two forms of such kinases, NFR1 (or LYK3 in *M. truncatula*) and NFR5 (or NFP in *M. truncatula*) [20, 24, 39]. In previous researches [27], peanut NFR1 was found to have incomplete LysM receptor-kinase domains. Moreover, the transcriptome of Nod- E4 and Nod+ E5 showed that *NFR1* was not a differentially expressed gene in any genotypes [26]. In this study, four putative *AhNFR1* genes (*AhNFR1A1*, *AhNFR1B1*, *AhNFR1A2* and *AhNFR1B2)* were identified in the peanut genome. The high homology of DNA sequences between *AhNFR1A1* and *AhNFR1B1*, and between *AhNFR1A2* and *AhNFR1B2* indicated that these genes might have similar functions. However, the sequence length of *AhNFR1A1* is longer than others, with two additional domains (STKc_IRAK and PKc_like superfamily) annotated in PeanutBase. A different *AhNFR1A1* gene structure (gene symbol: LOC112702229) was annotated in the NCBI database, which was shorter in sequence length. To keep the gene model consistent with Legume Information System, we used the gene information from PeanutBase in this study.

Among the four identified *AhNFR1* genes, *AhNFR1A2* and *AhNFR1B2* might be related to nodule formation since they were induced in Nod+ E5 but not in Nod- E4 after rhizobial infection. However, all 10 knockout events of *AhNFR1A2* and *AhNFR1B2* genes, produced nodules except two lines, which indicated that *AhNFR1A2* and *AhNFR1B2* may not be an absolutely required gene for nodule formation. Most likely, other *NFR1* paralogs complemented the function of tested *NFR1* copy for nodulation in peanut. *AhNFR1* genes were relatively divergent with many copies. Using the soybean *NFR1* gene sequence as a query, we blasted the peanut genomes and identified four copies with very stringent homology criteria. However, when we relaxed the criteria, many more *NFR1* like genes could be identified in peanut genomes. For example, when the E value was set at *e*^− 10^, 35 *NFR1* like genes can be identified. To identify which *NFR1* genes play roles in peanut nodule formation out of a large number of candidates is a big task. So, in this study we selected *AhNFR1A2* and *AhNFR1B2* genes for the knockout experiment. Further research should be conducted to validate the function of different copies of *NFR1* gene in peanut nodulation. An RNAi experiment could be a more valuable method to down-regulate all the paralogs efficiently in hairy roots than CRISPR/Cas9.

The LysM domain of NFR5 at least partially determines the specificity of NF recognition [26, 40]. NFR5 had been reported to be coupling with NFR1 as heterodimers in NF recognition signal transduction [29]. One *NFR5* ortholog (*AhNFP*) was identified in peanut genome with complete LysM receptor-kinase domains by Ibanez et al. [27]. In this study, two *AhNFR5* genes were identified with the same extracellular domain. DNA sequences of the two *AhNFR5* genes were the same between Nod- E4 and Nod+ E5 indicating that the different nodulation phenotypes between the sister recombinant inbred lines [26], E4 and E5, were not caused by mutation of *AhNFR5* genes. However, since *AhNFR5* gene was induced in Nod- E4, most likely the genes responsible for the different nodulation phenotypes between E4 and E5 are downstream of *AhNFR5* gene. Araip.NL2P7, one *NFR5* ortholog in diploid peanut (*Arachis ipaensis*), was identified as a differentially expressed gene (DEG) [26], which was up-regulated in Nod+ genotype in response to rhizobia inoculation, indicating the induction of its expression during the nodulation process. Here, the two *AhNFR5* genes had high expression levels in Nod+ genotype (E5) compared with Nod- genotype (E4), and also when both *AhNFR5A* and *AhNFR5B* were mutated in transgenic hairy roots, no nodule was formed. Therefore, we believe *AhNFR5* genes, either *AhNFR5A,* or *AhNFR5B,* or both are required for peanut nodule formation. Since it was confirmed that the lines with only *AhNFR5B* mutated had no nodule formation, at least *AhNFR5B* was required for peanut nodule formation. Whether *AhNFR5A* is required for nodule formation needs to be further explored. Similarly, in soybean, the mutants of either of the two *NFR5* genes showed Nod- phenotypes [22].

## Conclusions

The CRISPR/Cas9 in coupled with hairy root transformation system was established in peanut to study gene functions during nodule formation. The nodulation phenotype of mutants with editing in *AhNFR1* genes (*AhNFR1A2* and *AhNFR1B2* identified in this study) could still form nodules after rhizobia inoculation, whereas mutants with editing in *AhNFR5* genes (*AhNFR5A* and *AhNFR5B* identified in this study) showed Nod- phenotype. Yet, mosaic editing patterns detected for both genes may hinder the interpretation of their functions. These results showed that CRIPR/Cas9 system worked in allotetraploid peanut hairy roots can be used for preliminary genes screening. A stable embryogenic transformation CRISPR system should be applied to further confirm the genes’ function in the future.

## Methods

### Plant materials

Peanut cultivar ‘Tifrunner’ provided by Dr. Baozhu Guo at USDA-ARS (Tifton, GA) and a pair of peanut sister recombinant inbred lines (RILs), a non-nodulating (Nod-) ‘E4’and a nodulating (Nod+) ‘E5’ [26, 30] were used in this study. The growth condition of all the plants used in this study was 16-h-day 27 °C/8-h-night 25 °C cycle in growth chambers at University of Florida.

For gene expression analysis, approximately 200 seeds of each genotype were sterilized in 0.1% HgCl_2_ solution for seven minutes and then washed three times using sterilized ddH_2_O for five minutes each time. Seed germination and the rhizobia inoculation on peanut roots followed the method described by Peng et al. [26]. In brief, sterilized seeds were soaked in distilled water for 2 days, and then transferred into a germination box. After 4 days, germinated seeds were transferred to Ziploc bags with germination paper inserts containing 40 ml 25% Hoagland’s solution without N. A *Bradyrhizobium spp*. strain named Lb8 isolated from peanut nodules who had a high nodulation efficiency [41] was used for inoculum preparation. One ml of Lb8 (A_600_ = 0.05–0.1) suspension was applied to roots when they were 6–7 cm long. For each genotype, a total of 100 plants were inoculated with the Lb8. At 0, 2, 4, 6, 8, 16, 24, 48, 72, 96 and 144 h after inoculation (HAI), the middle 2–3 cm of the primary root was cut from treated plants and immediately put into liquid nitrogen for RNA extraction. The middle 2–3 cm of the peanut primary root at 6 days after germination harbors the active rhizobial infection sites, where the lateral roots are about to emerge [16, 17].

### Identification and characterization of *AhNFR* genes

To identify the genomic DNA sequences of *AhNFR1* and *AhNFR5* orthologs in peanut genomes, BLAST searches were performed by using the CDS of *GmNFR1* (DQ219806) and *GmNFR5* (NM_001354196) genes as queries against PeanutBase (https://peanutbase.org/) at E-value = 0. Domain search of AhNFR protein was performed by SWISS-MODEL homology modeling programs [42]. Phylogenetic analysis was conducted using MEGA 5 software [43]. Based on the CDS of the gene model from BLAST, we designed primers (Additional file 1: Table S3) to amplify them from the two genotypes, Nod- E4 and Nod+ E5, to obtain their cDNA sequences.

RNA samples were prepared from three biological replicates of both E4 and E5 at each time point after inoculation as described above. Total RNA was isolated using the Direct-zol RNA Miniprep Kit according to the manufacturer’s protocol (Zymo Research, USA). First strand cDNA synthesis was carried out using a high capacity RNA-to-cDNA Kit (Applied Biosystems, USA). Due to the high similarity between the DNA sequences of homoeologous copies, primers for q-PCR were carefully designed from the diverged sequence regions between the homoeologous copies to amplify homoeologous gene-specific copy. Primers’ (Additional file 1: Table S4) amplification efficiency (E) was estimated by running q-PCR of a serial of diluted cDNA samples (E4 at 0 HAI). The standard curve was constructed by plotting the log of the starting quantity of template against the Ct value obtained during amplification of each dilution. The E values were calculated from the slope of the standard curve using the following formula: E = 10^–1/slope^. Only the primers with E value in a range of 90–105% were used for q-PCR. q-PCR was performed with three biological replicates and three technical replicates for each sample using the Power SYBR Green PCR Master Mix kit (Applied Biosystems, USA) and CFX96 Real-Time PCR Detection System (Bio-Rad, USA). Results obtained from the different treatments were standardized to the *AhUbiquitin* 2 [44], an internal control for peanut q-PCR. Relative gene expression was calculated using the 2^−ΔΔCt^ method [45]. Melting curves were recorded after cycle 40 by heating from 55 to 95 °C. Statistical analysis of the gene expression level among different time points or between the two varieties was performed by applying one-way analysis of variance (ANOVA). Probability values of less than 0.05 were considered significant, and an asterisk or letter identifies such significance in Fig. 3.

### sgRNA design and construction of sgRNA: Cas9 expression vector

p201G/Cas9 plasmid (#59178) and pUC-gRNA shuttle plasmid (#47024) were ordered from Addgene (http://www.addgene.org/). The sgRNA cassette in pUC-gRNA was induced through the MtU6 promoter. Cas9 and GFP in p201G/Cas9 separately were driven by an enhanced cauliflower mosaic virus (CaMV) 35S promoter. GFP was used as a visible marker to rapidly screen transgenic events.

The sgRNAs in the constructed vectors were designed by using the web-based tool CRISPR-P (http://cbi.hzau.edu.cn/crispr/) [46], which highlighted all potential CRISPR sgRNA sequences (19 bp or 20 bp) immediately followed by 5′-NGG (PAM) in the forward or reverse strands of the *AhNFR* CDS. For each target locus we chose, DNA oligos were synthesized from Invitrogen (Carlsbad, USA). DNA oligos were inserted between mtU6 promotor and sgRNA scaffolds by using overlapping PCR. The overlapping PCR products were ligated into p201G/Cas9 plasmid vector which was digested by restriction enzymes *ApaI* and *SpeI* [34]. The constructs (p201G/Cas9: sgRNAs) were first transformed into *E. coli TOP10*. The PCR (Additional file 1: Table S5) positive clones of the constructs were purified using Plasmid Kit (NEB, England). After Sanger sequencing of the constructs at the cloning site, the successfully constructed plasmid was then selected and used to transform *Agrobacterium rhizogenes* strain K599.

### Peanut hairy root transformation

Hairy root transformation system using the *A. rhizogenes* strain K599 was described previously [47]. Briefly, in this study, K599 carrying the CRISPR/Cas9 plasmids of interest was grown on yeast mannitol plate supplemented with 50 μg/ml kanamycin (Sigma, USA) for 2 days. Tifrunner peanut seedlings at 7 days after germination (DAG) were injected with K599 at the hypocotyl. Each GFP positive hairy root is an independent transgenic event because every hairy root develops independently after k599 injection for hairy root induction. After being injected, the peanut seedlings were transferred into pots filled with vermiculite and grown in the growth chamber (16-h-day 27 °C/8-h-night 25 °C cycle).

### GFP examination, genomic DNA extraction and mutation analyses

Four weeks after transformation, the transformed peanut plants were dug out and observed under a stereomicroscope (Olympus, Japan). The plant seedlings with GFP positive hairy roots were replanted into vermiculite for recovery and then were inoculated by rhizobia strain Lb8 [26]. At 1 month after rhizobia inoculation, nodulation phenotype was observed. The DNA of GFP positive hairy roots were extracted using the CTAB method as previously described [48]. The target gene regions of the GFP positive hairy roots were amplified (Additional file 1: Fig. S2 and Table S6), and cloned into pClone007 simple vector (TsingKe, Nanjing, China). For each target region amplicon, 20 positive clones were selected for Sanger sequencing. The sequences were analyzed and aligned by using DNAman software (Lynnon Biosoft, USA).

## Supplementary information

**Additional file 1 Fig. S1**. DNA sequence homology of *AhNFR* genes in peanut. **Fig. S2**. Schematics of gene sequence, target sites and the regions of examined PCR products of *AhNFR* genes in transgenic hairy roots. (A) *AhNFR1A2* and *AhNFR1B2* genes, (B) *AhNFR5A* and *AhNFR5B* genes. NFR1AB1, NFR1AB2, NFR5AB1, NFR5AB2, and NFR5B are gRNAs. AhNFR1-EX-h, AhNFR5-EX-h, AhNFR5B-EX-h represent amplicons covering the target sites for examining the edited gene sequence. **Fig. S3.** PCR products of *AhNFR1* gene in transgenic hairy roots. M: 1 kb plus marker; H1-H12: transgenic *AhNFR1* samples; P1-P3: hairy roots with P201G empty vector; WT: Tifrunner peanut; −: negative control. **Fig. S4.** PCR products of *AhNFR5* gene in transgenic hairy roots. M: DL2000 marker; F1-F10: transgenic *AhNFR5* samples; P1-P3: hairy roots with P201G empty vector; WT: Tifrunner peanut; −: negative control. **Fig. S5.** PCR products of *AhNFR5B* gene in transgenic hairy roots. M: 1 kb plus marker; 5B1-5B10: transgenic *AhNFR5B* samples; P1-P3: hairy roots with P201G empty vector; WT: Tifrunner peanut; −: negative control. **Table S1.** Percentage of different CRISPR/Cas9-caused mutations at *AhNFR1A2 and AhNFR1B2* DNA sequence sites of transgenic *AhNFR1* hairy roots. **Table S2.** Percentage of different CRISPR/Cas9-caused mutations at *AhNFR5A and AhNFR5B* DNA sequence sites of transgenic *AhNFR5* hairy roots. **Table S3.** The primers of *AhNFR* genes. **Table S4.** The primers of q-PCR of *AhNFR* genes. **Table S5.** Primers for constructing and examining CRISPR/Cas9:*AhNFR* vectors. **Table S6.** Primers for amplifying the target genes of transgenic *AhNFR* hairy roots.

**Additional file 2.** Original gel images presented in Fig. S3, Fig. S4, and Fig. S5.

## Data Availability

The data generated or analyzed in this study are included in this article and supplementary information files. The sequencing data generated in this study were deposited in NCBI SRA database (BioProject: PRJNA655791, and SRA accession number: SRR12405032-SRR12405036). Other materials are available upon reasonable request to the corresponding author at wangjp@ufl.edu.

## Abbreviations

CDS: Coding DNA sequences
GFP: Green fluorescent protein
CRISPR/Cas9: Clustered regularly interspaced short palindromic repeats/CRISPR-associated protein 9
HAI: Hours after inoculation
LYK: LysM receptor-like kinases
LysM: Lysin motif
NF: Nod factor
NFP: NF perception
NFR: NF receptor
q-PCR: Quantitative real time-PCR
RNAi: RNA interference
sgRNAs: Single guide RNAs
