# Supplementary material for: The application of CRISPR/Cas9 in hairy roots to explore the functions of AhNFR1 and AhNFR5 genes during peanut nodulation
Source: BMC Plant Biol. 2020 Sep 7;20:417. doi: 10.1186/s12870-020-02614-x (PMC7487912; doi:10.1186/s12870-020-02614-x)
Supplement: Supplementary file 1 — Additional file 1 Fig. S1. DNA sequence homology of AhNFR genes in peanut. Fig. S2. Schematics of gene sequence, target sites and the regions of examined PCR products of AhNFR genes in transgenic hairy roots. (A) AhNFR1A2 and AhNFR1B2 genes, (B) AhNFR5A and AhNFR5B genes. NFR1AB1, NFR1AB2, NFR5AB1, NFR5AB2, and NFR5B are gRNAs. AhNFR1-EX-h, AhNFR5-EX-h, AhNFR5B-EX-h represent amplicons covering the target sites for examining the edited gene sequence. Fig. S3. PCR products of AhNFR1 gene in transgenic hairy roots. M: 1 kb plus marker; H1-H12: transgenic AhNFR1 samples; P1-P3: hairy roots with P201G empty vector; WT: Tifrunner peanut; −: negative control. Fig. S4. PCR products of AhNFR5 gene in transgenic hairy roots. M: DL2000 marker; F1-F10: transgenic AhNFR5 samples; P1-P3: hairy roots with P201G empty vector; WT: Tifrunner peanut; −: negative control. Fig. S5. PCR products of AhNFR5B gene in transgenic hairy roots. M: 1 kb plus marker; 5B1-5B10: transgenic AhNFR5B samples; P1-P3: hairy roots with P201G empty vector; WT: Tifrunner peanut; −: negative control. Table S1. Percentage of different CRISPR/Cas9-caused mutations at AhNFR1A2 and AhNFR1B2 DNA sequence sites of transgenic AhNFR1 hairy roots. Table S2. Percentage of different CRISPR/Cas9-caused mutations at AhNFR5A and AhNFR5B DNA sequence sites of transgenic AhNFR5 hairy roots. Table S3. The primers of AhNFR genes. Table S4. The primers of q-PCR of AhNFR genes. Table S5. Primers for constructing and examining CRISPR/Cas9:AhNFR vectors. Table S6. Primers for amplifying the target genes of transgenic AhNFR hairy roots. [file 12870_2020_2614_MOESM1_ESM.docx]

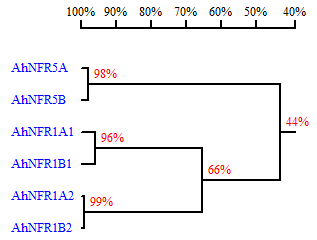


**Figure S1. DNA sequence homology of *AhNFR* genes in peanut.**


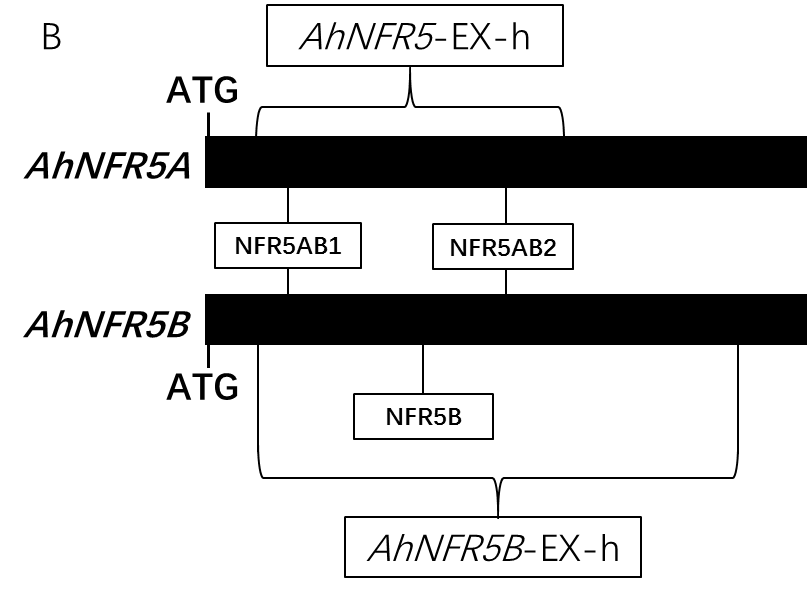

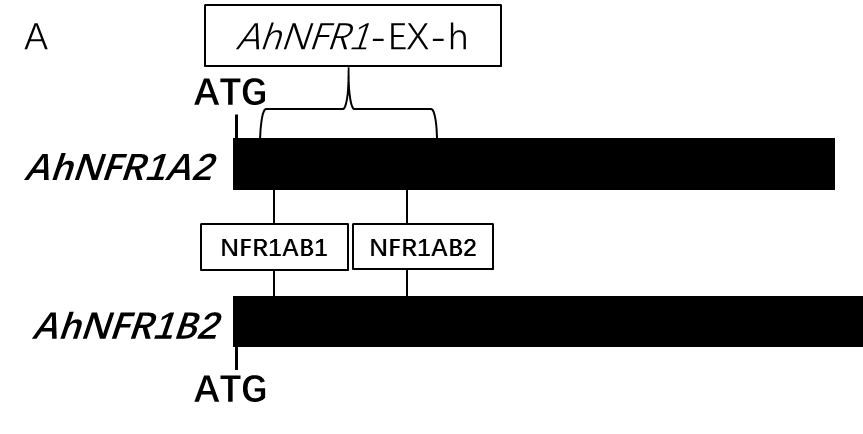


**Figure S2. Schematics of gene sequence, target sites and the regions of examined PCR products of *AhNFR* genes in transgenic hairy roots.** (A) *AhNFR1A2* and *AhNFR1B2* genes, (B) *AhNFR5A* and *AhNFR5B* genes. NFR1AB1, NFR1AB2, NFR5AB1, NFR5AB2, and NFR5B are gRNAs. AhNFR1-EX-h, AhNFR5-EX-h, AhNFR5B-EX-h represent amplicons covering the target sites for examining the edited gene sequence.

**Figure S3. PCR products of *AhNFR1* gene in transgenic hairy roots.**

M: 1kb plus marker; H1-H12: transgenic *AhNFR1* samples; P1-P3: hairy roots with P201G empty vector; WT: Tifrunner peanut; -: negative control.

**Figure S4. PCR products of *AhNFR5* gene in transgenic hairy roots.**

M: DL2000 marker; F1-F10: transgenic *AhNFR5* samples; P1-P3: hairy roots with P201G empty vector; WT: Tifrunner peanut; -: negative control.

**Figure S5. PCR products of *AhNFR5B* gene in transgenic hairy roots.**

M: 1kb plus marker; 5B1-5B10: transgenic *AhNFR5B* samples; P1-P3: hairy roots with P201G empty vector; WT: Tifrunner peanut; -: negative control.

**Table S1. Percentage of different CRISPR/Cas9-caused mutations at *AhNFR1A2 and AhNFR1B2* DNA sequence sites of transgenic *AhNFR1* hairy roots.**

| **Samples** | **Genes** | **Different nucleotide deletion (-) and insertion (+) events** | | | | | | | | | | | | |
| --- | --- | --- | --- | --- | --- | --- | --- | --- | --- | --- | --- | --- | --- | --- |
|  |  | **InDel type1** | **%** | **InDel type2** | **%** | **InDel type3** | **%** | **InDel type4** | **%** | **InDel type5** | **%** | **InDel type6** | **%** |  |
| H1-L | *NFR1A2* | 0,0 | **39** | -2,-2 | **33** | -2,-1 | **28** |  |  |  |  |  |  |  |
|  | *NFR1B2* | 0,0 | **100** |  |  |  |  |  |  |  |  |  |  |  |
| H2-L | *NFR1A2* | 0,0 | **5** | -10,-1 | **58** | +1,-1 | **32** | +1,-5 | **5** |  |  |  |  |  |
|  | *NFR1B2* | 0,0 | **100** |  |  |  |  |  |  |  |  |  |  |  |
| H3-L | *NFR1A2* | -9,-1 | **58** | -28,-1 | **25** | -10,-1 | **8** | 0,-1 | **8** |  |  |  |  |  |
|  | *NFR1B2* | -28,-1 | **100** |  |  |  |  |  |  |  |  |  |  |  |
| H4-L | *NFR1A2* | 0,0 | **67** | -10,-1 | **33** |  |  |  |  |  |  |  |  |  |
|  | *NFR1B2* | +1,-1 | **100** |  |  |  |  |  |  |  |  |  |  |  |
| H5-L | *NFR1A2* | -10,-1 | **42** | +1,-1 | **17** | 0,-1 | **17** | -12,-1 | **8** | -13,-1 | **8** | -22,-1 | **8** |  |
|  | *NFR1B2* | -10,-1 | **25** | 0,-1 | **25** | -7,-1 | **13** | -3,-1 | **13** | +1,-1 | **13** | +3,-1 | **13** |  |
| H6-L | *NFR1A2* | -2,-1 | **33** | -38,-1 | **33** | -10,-1 | **11** | -12,-1 | **11** | -7,-1 | **11** |  |  |  |
|  | *NFR1B2* | -28,-9 | **27** | -7,-1 | **18** | -6,0 | **9** | -7,-12 | **9** | 0,-3 | **9** | -1/-13/-37,0 | **27** |  |
| H7-L | *NFR1A2* | -7,-1 | **27** | 0,0 | **27** | +1,-5 | **9** | +1,-1 | **9** | 0,-1 | **9** | -9/-2,-1 | **18** |  |
|  | *NFR1B2* | 0,0 | **33** | -2,-1 | **44** | -15,0 | **22** |  |  |  |  |  |  |  |
| H8-L | *NFR1A2* | -4,-6 | **100** |  |  |  |  |  |  |  |  |  |  |  |
|  | *NFR1B2* | -41,-6 | **82** | -41,0 | **12** | -13,-1 | **6** |  |  |  |  |  |  |  |
| H9-L | *NFR1A2* | **N.A.** |  |  |  |  |  |  |  |  |  |  |  |  |
|  | *NFR1B2* | -4,-6 | **45** | -4,-3 | **55** |  |  |  |  |  |  |  |  |  |
| H10-L | *NFR1A2* | -9,-1 | **11** | -3,-72 | **11** | -3/0,+1 | **22** | +1,-57 | **11** | -10/0,-1 | **11** | -3/-7,-2 | **22** |  |
|  | *NFR1B2* | -4,-1 | **73** | +1,-5 | **9** | 0,-57 | **9** | -4,-13 | **9** |  |  |  |  |  |

H1-L~H10-L: alignment of genomic sequences cloned from the long PCR products using DNA samples H1~H10 of transgenic *AhNFR1* hairy roots as templates. InDel type shows the type of insertion (+), or deletion (-). The number following the + or - are the inserted (+) or deleted (-) number of nucleotides. ‘%’ represents percentage of the type of InDel among all edit events detected in the sample.

**Table S2.** **Percentage of different CRISPR/Cas9-caused mutations at *AhNFR5A and AhNFR5B* DNA sequence sites of transgenic *AhNFR5* hairy roots.**

| **Samples** | **Genes** | **Different nucleotide deletion (-) and insertion (+) events** | | | | | | | |
| --- | --- | --- | --- | --- | --- | --- | --- | --- | --- |
|  |  | **InDel type1** | **%** | **InDel type2** | **%** | **InDel type3** | **%** | **InDel type4** | **%** |
| F1 | *NFR5A* | -5,-4 | **38** | -5,-5 | **25** | -5,0 | **25** | -1,-5 | **13** |
|  | *NFR5B* | -3,0 | **25** | -11,-7/-3 | **33** | -3,-3/-8 | **25** | 0,+1 | **17** |
| F2 | *NFR5A* | 0,0 | **50** | -2,-7 | **17** | -4,-7 | **17** | -1,-8 | **17** |
|  | *NFR5B* | 0,0 | **50** | +1,-3 | **25** | -1,-3 | **13** | -1,-7 | **13** |
| F3 | *NFR5A* | -7,-2 | **58** | +1,0 | **25** | +1,-2 | **8** | -1,0 | **8** |
|  | *NFR5B* | -5,0 | **86** | +1,-7 | **14** |  |  |  |  |
| F4 | *NFR5A* | 0,0 | **100** |  |  |  |  |  |  |
|  | *NFR5B* | 0,0 | **83** | -5,0 | **17** |  |  |  |  |
| F5 | *NFR5A* | 0,0 | **67** | -5,0 | **17** | +1,0 | **17** |  |  |
|  | *NFR5B* | 0,0 | **21** | -4,-13 | **64** | -5,0 | **7** | 0,-13 | **7** |
| F6-L | *NFR5A* | -1,0 | **100** |  |  |  |  |  |  |
|  | *NFR5B* | **N.A.** |  |  |  |  |  |  |  |
| F6-S | *NFR5A* | -736 | **100** |  |  |  |  |  |  |
|  | *NFR5B* | **N.A.** |  |  |  |  |  |  |  |
| F7 | *NFR5A* | 0,0 | **100** |  |  |  |  |  |  |
|  | *NFR5B* | 0,0 | **100** |  |  |  |  |  |  |
| F8 | *NFR5A* | 0,0 | **20** | -1,0 | **80** |  |  |  |  |
|  | *NFR5B* | 0,0 | **80** | -16,0 | **20** |  |  |  |  |
| F9 | *NFR5A* | +1,0 | **80** | -34,-5 | **20** |  |  |  |  |
|  | *NFR5B* | -3,-2 | **30** | -4,-2 | **30** | +1,0 | **20** | -4,-50 | **20** |
| F10 | *NFR5A* | 0,0 | **100** |  |  |  |  |  |  |
|  | *NFR5B* | 0,0 | **100** |  |  |  |  |  |  |

F1~F10: alignment of genomic sequences cloned from the PCR products using DNA samples F1~F10 of transgenic *AhNFR5* hairy roots as templates (F6-L/F6-S represent clones are from the long or short PCR products respectively.). InDel type shows the type of insertion (+) or deletion (-). The numbers following the + or - are the inserted (+) or deleted (-) number of nucleotides. ‘%’ represents percentage of the type of InDel among all edit events detected in the sample.

**Table S3. The primers of *AhNFR* genes.**

|  | **Forward primer** | **Reverse primer** |
| --- | --- | --- |
| *AhNFR1A2* | ATGGAACCCAGACATAGG | CTATCTTCCGGACATGAG |
| *AhNFR1B2* | ATGGAACCCAGATATGGG | CTATCTTCCGGACATGAG |
| *AhNFR5A* | ATGGCTTTCTTTCTACCCTCT | TTAACGTGCTGCTATGGGAGT |
| *AhNFR5B* | ATGGCTTTCTTTCTACCC | TTAACGTGCTGCTATGGGA |
|  | | |

**Table S4. The primers of q-PCR of *AhNFR* genes.**

|  | **Forward primer** | **Reverse primer** |
| --- | --- | --- |
| *AhNFR1A1-q-PCR* | ACAGAGGCAGATCCGAAGCG | GAAGGAAGCAATGTCCCAAT |
| *AhNFR1B1-q-PCR* | AGTTTGCCCTGGTTTCTGC | CAAGATTCTATTTCCTCCGTCC |
| *AhNFR1A2-q-PCR* | GTTTGTATCTGTGCTCTTGCAG | AGTTGACTATGTCGTTTGGGTC |
| *AhNFR1B2-q-PCR* | TCCCTAACACTGGAACCCTT | CACTCTGATTCTTCGCCACA |
| *AhNFR5A-q-PCR* | CTCATTGAGTTGCTGACAGGC | CCATCTTCTCAGACACTCCTCC |
| *AhNFR5B-q-PCR* | ATGTTCTCCATCACCAGCATC | TGTTCCACAGGAAGAAGGTGA |

**Table S5. Primers for constructing and examining CRISPR/Cas9:*AhNFR* vectors.**

|  | **Forward primer** | **Reverse primer** |
| --- | --- | --- |
| Primer-EX-V | CCCACATCGTCTGAAACA | GTCATCCACCTTAGCCATC |
|  |  |  |
| SpeI_MtU6-Forward | CGTGCTCCACCATGTTGGGAATGCCTATCTTATATGATCAATGAGG | |
| MtU6-Reverse | AAGCCTACTGGTTCGCTTGAAG | |
| Scaffold- Forward | GTTTTAGAGCTAGAAATAGCAAGTT | |
| ApaI_Scaffold- Reverse | GTGCTCCACCATGTTGGGCCAAAAAAAGCACCGACTCGGTG | |
| UNS1_Scaffold- Reverse | GAGAATGGATGCGAGTAATGAAAAAAAGCACCGACTCGGTG | |
| UNS1_MtU6- Forward | CATTACTCGCATCCATTCTCATGCCTATCTTATATGATCAATGAGG | |

**Table S6. Primers for amplifying the target genes of transgenic *AhNFR* hairy roots.**

|  | **Forward primer** | **Reverse primer** |
| --- | --- | --- |
| AhNFR1-EX-h | TTGCTGCTGGGTTGCGTTCTTTA | GAGCCTTGGTTGAAATTGACAGA |
| AhNFR5-EX-h | TTCTTGAGCCTTTCTAACA | AAGGGACCCGTTATCAGC |
| AhNFR5B-EX-h | ACATACATCGCTAAATCTCCAAAC | CATCTTCTCAGACATTCCTCCTT |
