# Supplementary material for: The application of CRISPR/Cas9 in hairy roots to explore the functions of AhNFR1 and AhNFR5 genes during peanut nodulation
Source: BMC Plant Biol. 2020 Sep 7;20:417. doi: 10.1186/s12870-020-02614-x (PMC7487912; doi:10.1186/s12870-020-02614-x)
Supplement: Supplementary file 2 — Additional file 2. Original gel images presented in Fig. S3, Fig. S4, and Fig. S5. [file 12870_2020_2614_MOESM2_ESM.docx]

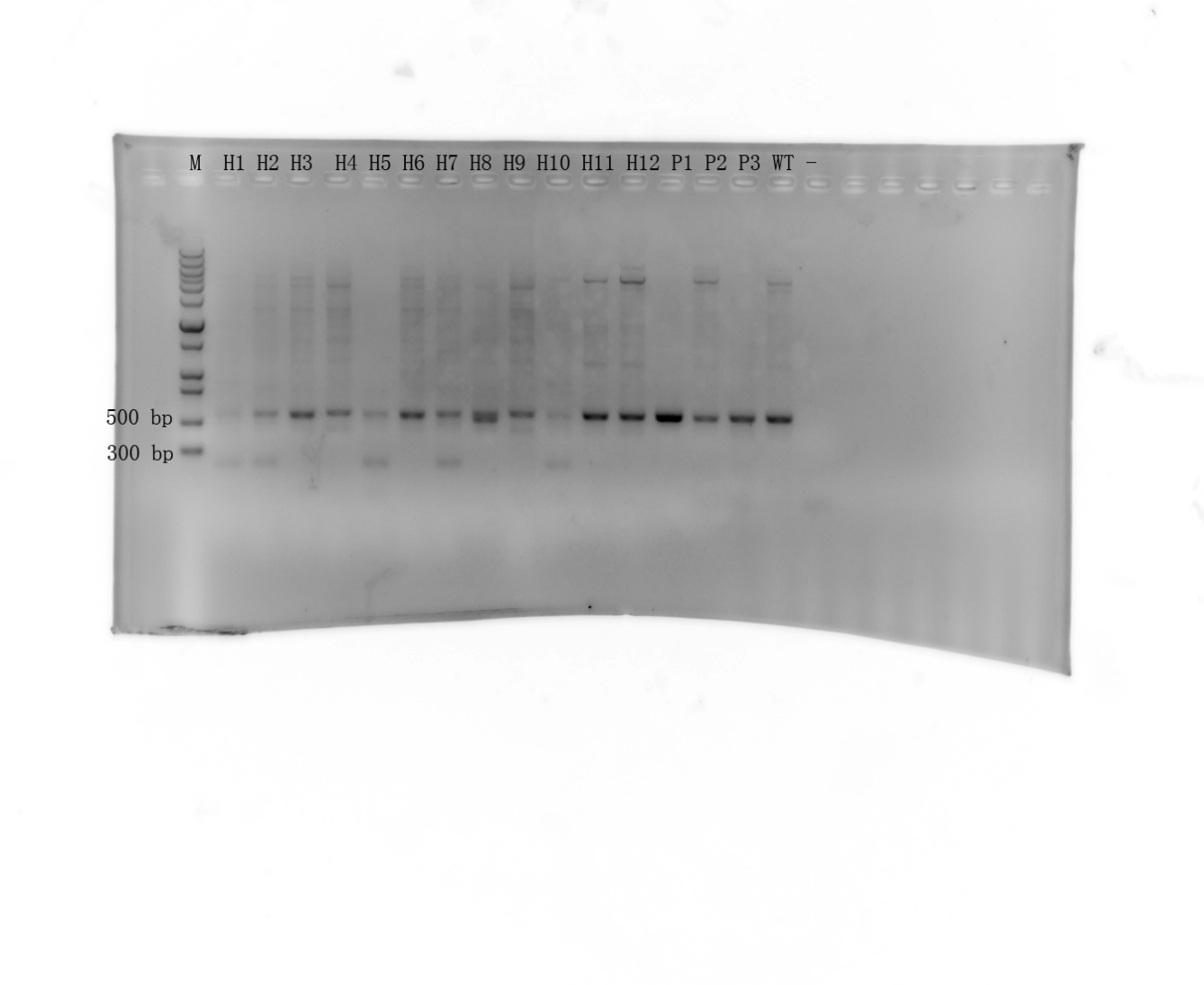


**Figure S3. PCR products of *AhNFR1* gene in transgenic hairy roots.**

M: 1kb plus marker; H1-H12: transgenic *AhNFR1* samples; P1-P3: hairy roots with P201G empty vector; WT: Tifrunner peanut; -: negative control.


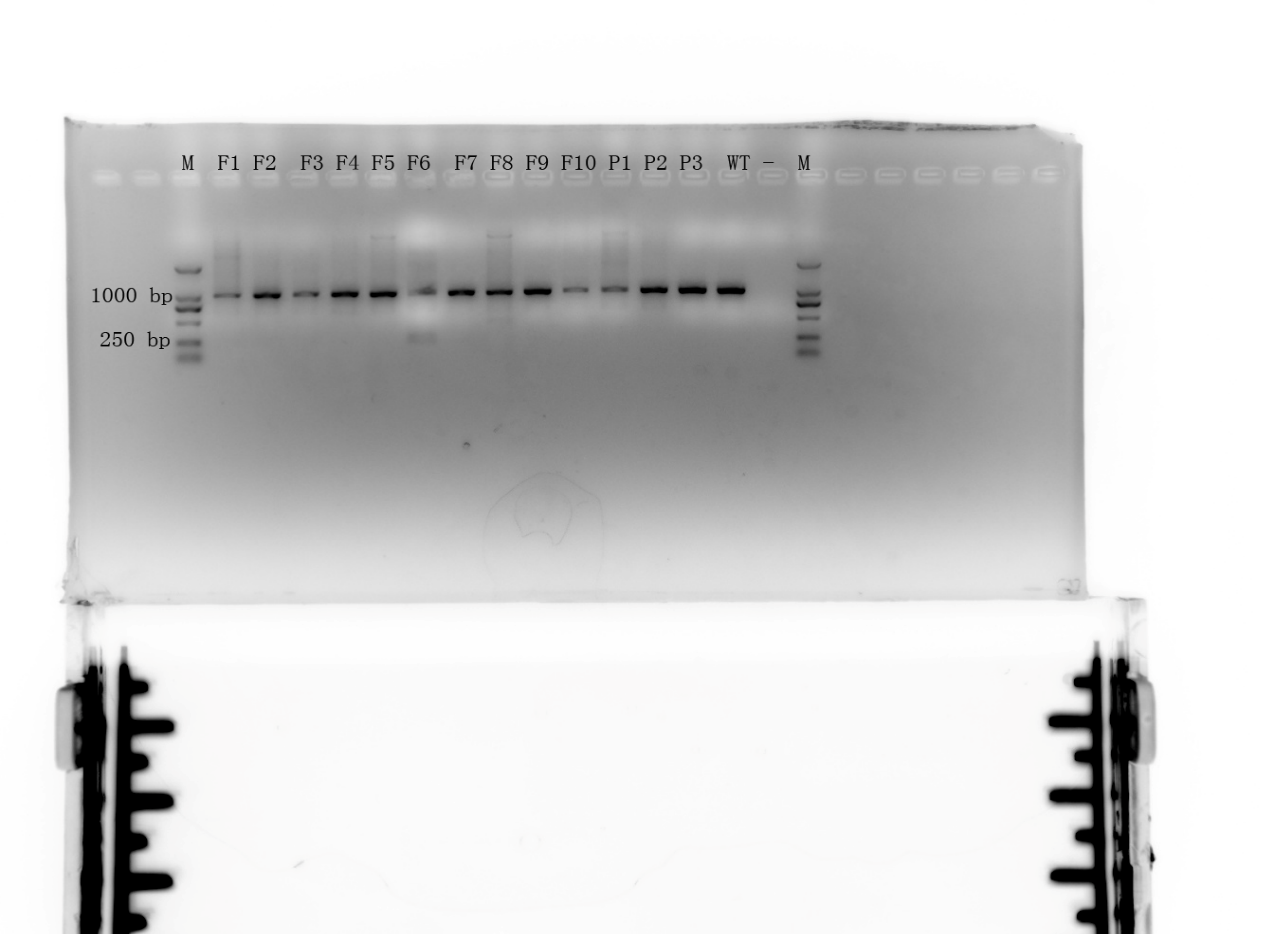


**Figure S4. PCR products of *AhNFR5* gene in transgenic hairy roots.**

M: DL2000 marker; F1-F10: transgenic *AhNFR5* samples; P1-P3: hairy roots with P201G empty vector; WT: Tifrunner peanut; -: negative control.


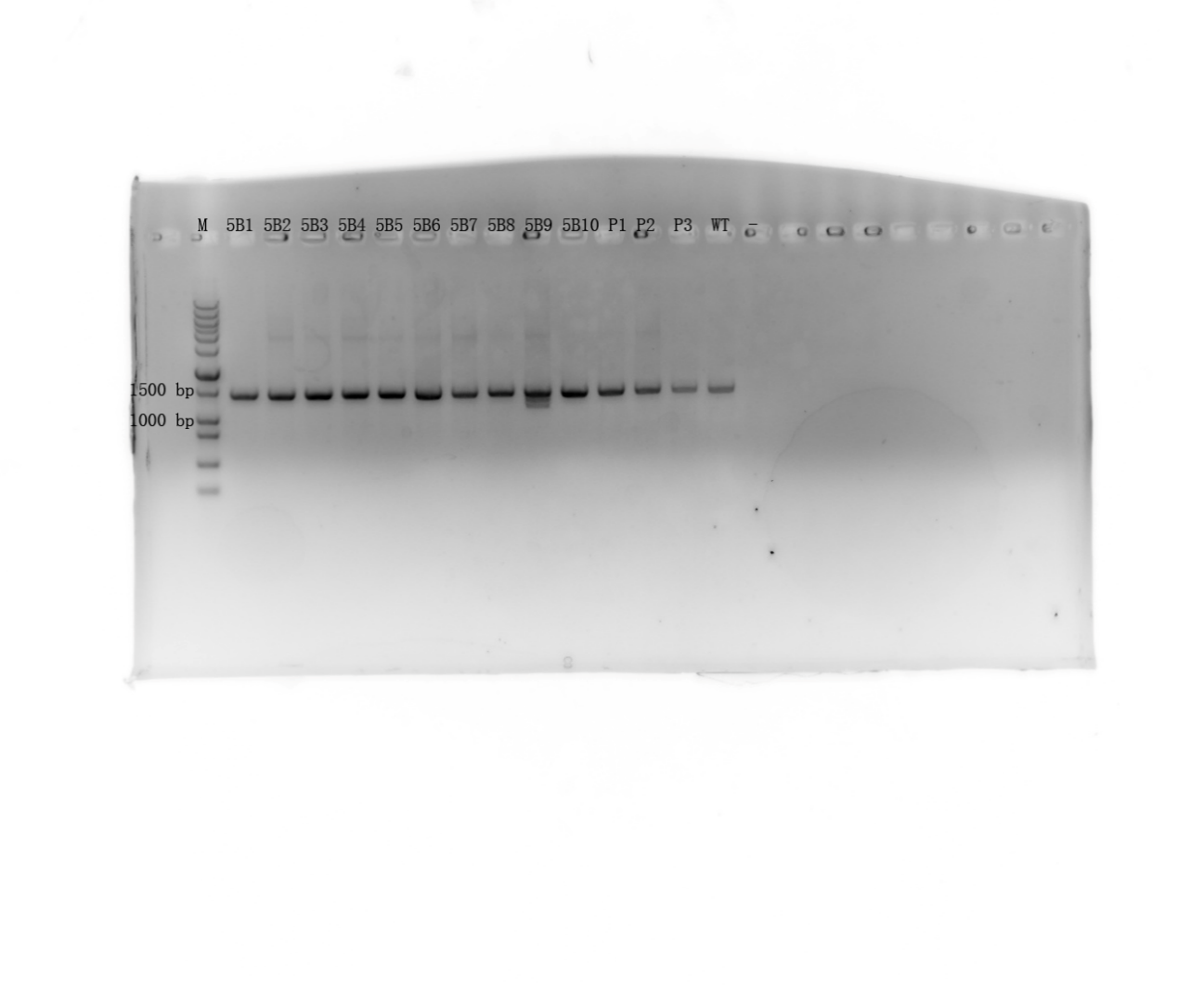


**Figure S5. PCR products of *AhNFR5B* gene in transgenic hairy roots.**

M: 1kb plus marker; 5B1-5B10: transgenic *AhNFR5B* samples; P1-P3: hairy roots with P201G empty vector; WT: Tifrunner peanut; -: negative control.
